# Obturator Nerve Injury: An Infrequent Complication of TOT Procedure

**DOI:** 10.1155/2014/290382

**Published:** 2014-09-29

**Authors:** S. Aydogmus, S. Kelekci, H. Aydogmus, E. Ekmekci, Y. Secil, S. Ture

**Affiliations:** ^1^Department of Obstetrics and Gynaecology, School of Medicine, İzmir Katip Çelebi University, Karabaglar, 35150 Izmir, Turkey; ^2^Department of Gynaecology and Obstetrics, Ataturk Research and Training Hospital, İzmir Katip Çelebi University, Karabaglar, 35150 Izmir, Turkey; ^3^Department of Neurology, Ataturk Research and Training Hospital, İzmir Katip Çelebi University, Karabaglar, 35150 Izmir, Turkey; ^4^Department of Neurology, School of Medicine, İzmir Katip Çelebi University, Karabaglar, 35150 Izmir, Turkey

## Abstract

Transvaginal mid-urethral slings have become the most preferred surgical treatment option for female stress urinary incontinence. However, various complications have been reported for these operations occurring especially during penetration of the retropubic space. It can negatively affect patient's quality of life. Early treatment increases the chance of complete normalization of the functions. In this case report we presented a case of obturator nerve damage that was diagnosed and treated at early stage after TOT operation.

## 1. Introduction

Stress urinary incontinence is a major public health problem affecting 20% of women and impairing quality of life. Due to their efficacy, safety, and ease of application, transvaginal midurethral slings have become the most preferred surgical treatment option [1]. However, various complications have been reported of these operations occurring especially during the penetration of the retropubic space. Although the majority of complications are minor complications like bladder perforation, such as complications like vascular or bowel injury, nerve injury, hematoma development are possible complications that may be fatal. In order to reduce these complications, as an alternative method, transobturator tape (TOT) method has been developed by Delorme [2]. However, the TOT method is not a risk-free method and such complications like infection, erosion, and myositis have been reported in the literature [3].

It was reported that at 5% of cases have a leg pain and it is improved in one month with an analgesic therapy [4]. Nerve injury was reported in 0.7–0.9/1000 after midurethral sling surgery [5]. In this case report, we presented a case of obturator nerve damage that was diagnosed and treated at early stage after TOT operation.

## 2. Case Report

**Figure 1:**
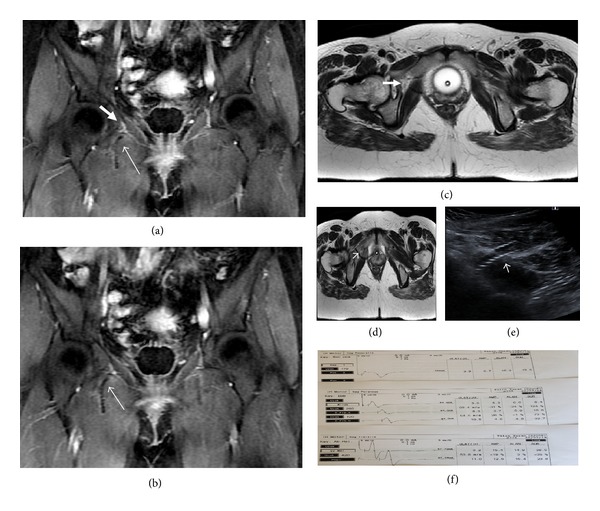


The patient was referred to us because of pain at the right leg, limitation, and inability to walk at the second postoperative day after midurethral sling (Safyre, Promedon) surgery which was performed for stress urinary incontinence. When the patient was admitted to the hospital, it was noted that adduction of the thigh was impaired. She was complaining about inability of adduction and paresthesias on the right thigh and she was not able to walk independently because of the loss of motor strength. These right adductor muscle symptoms were thought to be obturator nerve palsy. In magnetic resonance imaging (MRI) at coronal section, tape (thin arrow) was observed passing very close to the obturator bundle (thick arrow) at the right obturator fossa (Figures 1(a)-1(b)). This was confirmed at axial section (Figures 1(c)-1(d)). In pelvic ultrasonography, we did not detect edema or hematoma around tape (Figure 1(e)). Methylprednisolone, 48 mg, niacin, 250 mg, and pyridoxine, 250 mg per day, were given to the patient. At postoperative day five, cystoscopy was performed because symptoms were persisting. In cystoscopy, bladder and urethra were viewed intact. The present TOT sling was removed and a new minisling was performed at the same session. The patient's symptoms dramatically declined at the first postoperative day. Limitation in the flexion and adduction regressed and leg pain is significantly reduced at the right leg. The patient began to walk with assistance. She was discharged on postoperative day three without any problems. To clarify obturator nerve palsy, electrophysiological investigation (ENG-EMG) was performed. No pathological findings were observed in the first electromyography, which was performed on the 19th day of operation, and posterior tibial and fibular motor nerves and sural sensory nerve were all normal (Figure 1(f)). Electrophysiological findings in femoral innervated muscles and obturator innervated muscles were in normal limits. Partial or total axonal degeneration of a nerve cannot be detected up to 3 weeks electrophysiologically. By using this knowledge, the neurologist who performed the electromyographic investigation needed a second examination to clarify axonal degeneration of right obturator nerve. In the second ENG-EMG investigation, in the 6th week, pseudomyotonia, fibrillation potentials, and positive sharp waves were observed in the right adductor magnus muscle in needle EMG meaning partial axonal degeneration of the right obturator nerve. Electrophysiological findings in all other nerves and muscles in the right leg were normal again (posterior tibial nerve, fibular nerve, femoral nerve, and sural nerve). This obturator nerve axonal degeneration was compatible with clinical findings of the patient.

## 3. Discussion

Although transobturator route is adopted more securely than the retropubic passage, by both transvaginal midurethral sling methods, significant complications have been shown. According to data reported to the system by Manufacturer and User Facility Device Experience Database (MAUDE) in 2004, two neuropathy cases were present in reported 89 TOT related complications [6]. Obturator nerves are mixed sensory-motor nerves formed by L2-L4 spinal nerve roots. They innervate medial cutaneous skin of the thigh and leg, adductor muscles of leg, and proprioceptors of hip and knee joints. After exiting the spinal cord, it lies on the psoas muscle and passes through the minor pelvis. At the pelvic sidewall, it lies anteroinferiorly and leaves the pelvis by passing through the obturator foramen [7]. Obturator nerve injury may also develop during obstructed labor or the use of forceps. Also it can result after obturator hernia repair, TVT or TOT procedures, and hip surgeries [8, 9]. Clinically radiating pain, exacerbated with internal rotation and extension that is localized anteroinferior to the inguinal region and thigh, is in the foreground. On examination, paresthesia or hypoesthesia and loss of motor function in the adductor muscles may be viewed. Diagnosis is usually based on clinical findings. Denervation findings in electromyography (EMG) are not more specific. Computed tomography (CT) and magnetic resonance imaging (MRI) are helpful only in situations such as tumor, hematoma that cause mass effect. Decline of symptoms by infiltration of local anesthetic to the area is an effective method that can be used to confirm the diagnosis [8, 10].

It is not offered to replace a new mesh at management, after mesh excision due to mesh erosion. But it can be accepted at cases of unsuccessful results without mesh erosion or for relapse cases [7]. In our case, we have replaced a new minisling at the second surgery because mesh erosion was not present. We preferred minisling for less complication.

If conservative methods of treatment such as the obturator nerve block for relief of symptoms are insufficient, surgical exploration and primary nerve repair or grafting can be applied. Early treatment of injured obturator nerve often results in complete motor recovery as in our patient [11, 12]. Conversely, unless immediate repair is made, functional recovery on sensory and motor nerves is quite poor.

## 4. Conclusion

Obturator nerve injury is an infrequent complication of transvaginal midurethral sling operation. It can lead to symptoms like pain, paresthesia, and limitation in motor functions that negatively affect quality of life. Early treatment increases the chance of the complete normalization of the functions.

## References

[1] Bemelmans BLH, Chapple CR (2003). Are slings now the gold standard treatment for the management of female urinary stress incontinence and if so which technique?. *Current Opinion in Urology*.

[2] Delorme E (2001). Transobturator urethral suspension: a minimally invasive procedure to treat female stress urinary incontinence. *Progres en Urologie*.

[3] Álvarez-Bandrés S, Hualde-Alfaro A, Jiménez-Calvo J (2010). Complications of female urinary incontinence surgery with mini-sling system. *Actas Urologicas Espanolas*.

[4] Meschia M, Bertozzi R, Pifarotti P (2007). Peri-operative morbidity and early results of a randomised trial comparing TVT and TVT-O. *International Urogynecology Journal and Pelvic Floor Dysfunction*.

[5] Kuuva N, Nilsson CG (2002). A nationwide analysis of complications associated with the tension-free vaginal tape (TVT) procedure. *Acta Obstetricia et Gynecologica Scandinavica*.

[6] Novara G, Artibani W, Barber MD (2010). Updated systematic review and meta-analysis of the comparative data on colposuspensions, pubovaginal slings, and midurethral tapes in the surgical treatment of female stress urinary incontinence. *European Urology*.

[7] Costantini E, Lazzeri M, Porena M (2007). Managing complications after midurethral sling for stress urinary incontinence. *EAU-EBU Update Series*.

[8] Stewart JD (1993). *Focal Peripheral Neuropathies*.

[9] van Ba OL, Wagner L, de Tayrac R (2014). Obturator neuropathy: an adverse outcome of a trans-obturator vaginal mesh to repair pelvic organ prolapse. *International Urogynecology Journal and Pelvic Floor Dysfunction*.

[10] Corona R, de Cicco C, Schonman R, Verguts J, Ussia A, Koninckx PR (2008). Tension-free vaginal tapes and pelvic nerve neuropathy. *Journal of Minimally Invasive Gynecology*.

[11] Appell RA, Davila GW (2007). Treatment options for patients with suboptimal response to surgery for stress urinary incontinence. *Current Medical Research and Opinion*.

[12] Hong Y, O’Grady T, Lopresti D, Carlsson C (1996). Diagnostic obturator nerve block for inguinal and back pain: a recovered opinion. *Pain*.

